# De novo transcriptome sequencing and anthocyanin metabolite analysis reveals leaf color of *Acer pseudosieboldianum* in autumn

**DOI:** 10.1186/s12864-021-07715-x

**Published:** 2021-05-25

**Authors:** Yu-Fu Gao, Dong-Hui Zhao, Jia-Qi Zhang, Jia-Shuo Chen, Jia-Lin Li, Zhuo Weng, Li-Ping Rong

**Affiliations:** Agriculture College, Yanbian University, 977 Gongyuan Road, 133002 Yanji, China

**Keywords:** *A. pseudosieboldianum*, Transcriptome, Differentially expressed genes, Anthocyanin

## Abstract

**Background:**

Leaf color is an important ornamental trait of colored-leaf plants. The change of leaf color is closely related to the synthesis and accumulation of anthocyanins in leaves. *Acer pseudosieboldianum* is a colored-leaf tree native to Northeastern China, however, there was less knowledge in *Acer* about anthocyanins biosynthesis and many steps of the pathway remain unknown to date.

**Results:**

Anthocyanins metabolite and transcript profiling were conducted using HPLC and ESI-MS/MS system and high-throughput RNA sequencing respectively. The results demonstrated that five anthocyanins were detected in this experiment. It is worth mentioning that Peonidin O-hexoside and Cyanidin 3, 5-O-diglucoside were abundant, especially Cyanidin 3, 5-O-diglucoside displayed significant differences in content change at two periods, meaning it may be play an important role for the final color. Transcriptome identification showed that a total of 67.47 Gb of clean data were obtained from our sequencing results. Functional annotation of unigenes, including comparison with COG and GO databases, yielded 35,316 unigene annotations. 16,521 differentially expressed genes were identified from a statistical analysis of differentially gene expression. The genes related to leaf color formation including PAL, ANS, DFR, F3H were selected. Also, we screened out the regulatory genes such as MYB, bHLH and WD40. Combined with the detection of metabolites, the gene pathways related to anthocyanin synthesis were analyzed.

**Conclusions:**

Cyanidin 3, 5-O-diglucoside played an important role for the final color. The genes related to leaf color formation including PAL, ANS, DFR, F3H and regulatory genes such as MYB, bHLH and WD40 were selected. This study enriched the available transcriptome information for *A. pseudosieboldianum* and identified a series of differentially expressed genes related to leaf color, which provides valuable information for further study on the genetic mechanism of leaf color expression in *A. pseudosieboldianum*.

**Supplementary Information:**

The online version contains supplementary material available at 10.1186/s12864-021-07715-x.

## Background

Leaf color is one of the most important characteristics of ornamental plants, and plants with colored foliage were often called “colored-leaf plants” [1, 2]. Some researchers have analyzed and determined systematically the pigments and physiological indexes of the leaves of colored-leaf plants [3, 4]. Result showed that the change of leaf color is closely related to the synthesis and accumulation of anthocyanins in leaves [5]. Anthocyanins are one of the important secondary metabolites of plants and they often have anti-cancer, anti-oxidation and anti-atherosclerosis properties [6]. Anthocyanins confer orange, red, magenta, violet and blue and the biosynthetic pathway leading to floral or pulp pigment accumulation had been well characterized and the genes encoding relevant enzymes and transcriptional factors have been isolated [7, 8]. The molecular mechanisms of the anthocyanin biosynthesis pathway also have been comprehensively reported. However, most of the researches mainly focused on f fruit color [9] and petal color [10–12], and anthocyanin biosynthesis in colored-leaf plants has rarely been researched prior to this study. In recent years, some scholars have identified PAL, CHS, CHI, DFR, ANS, F3H, F3’H, F3’5’H [13, 14] and a few related regulatory genes such as MYB, bHLH and WDR in color changing of colored-leaf plants [15, 16]. The process of anthocyanin synthesis and accumulation is relatively complex, and is regulated by multiple enzymes and transcription factors [17], as well as being influenced by external environmental factors such as light [18], water stress [19], and temperature [20]. Thus the mechanism of leaf color change in colored-leaf plants needs to be further studied.

*Acer pseudosieboldianum* is a small deciduous tree belonging to the *Acer* genus of the family Aceraceae. Because of its beautiful shape and brilliant leaves, it is an often used autumn leaf ornamental tree species [21]. In addition, it has high economic value, whose woods can be used for making utensils and leaves can be used as dyes [22]. Recently, some scholars have reported and studied the introduction, cultivation, and breeding of *A. pseudosieboldianum* [23, 24]. However the key genes affecting leaf color change have not been determined yet, and relative information is relatively scarce. This fact means that the molecular regulatory mechanisms related to leaf color formation needs further study.

In recent years, transcriptome high-throughput sequencing technology has been widely used to study the mechanism of leaf color in various plants [25, 26]. In this study, de novo transcriptome sequencing assembly, annotation, and bioinformatic analysis on leaves from *A. pseudosieboldianum* were performed at different color-changing stages in autumn. The DEGs at different transformation stages were analyzed and validated. At last, combined this data with anthocyanin metabolism analysis data, some genes related to anthocyanin synthesis were identified. This study provides a theoretical basis for studying the molecular mechanism of leaf color in *A. pseudosieboldianum*.

## Results

### Contents of anthocyanin in the leaves

**Fig. 1:**
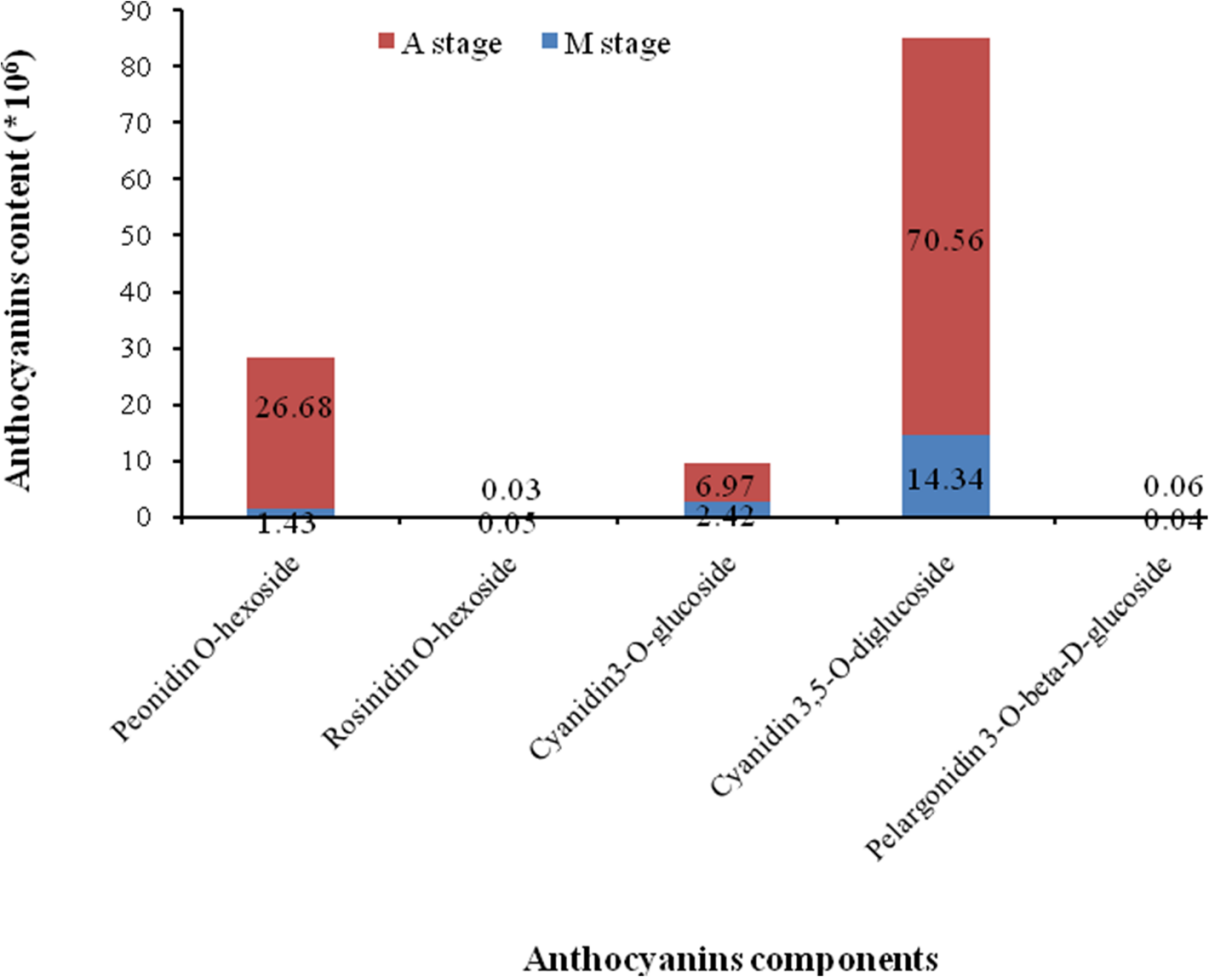
Anthocyanins components and contents detected in *A. pseudosieboldianum*

In order to explore the mechanism of pigment formation in A. pseudosieboldianum leaves, we carried out qualitative analysis of anthocyanin components in the middle (M) and last stage (A) of leaf color transformation (The anthocyanin content was extremely low in early stage (B), Therefore, only M and A stage were analyzed). According to our UPLC–Q–TOF–MS data, five anthocyanins were identified (Fig. 1). They were Peonidin O-hexoside, Rosinidin O-hexoside, Cyanidin 3-O-glucoside, Cyanidin 3, 5-O-diglucoside, and Pelargonidin 3-O-beta-D-glucoside. The content of five anthocyanin metabolites were different during the middle stage (M) and last stage (A). The contents of Rosinidin O-hexoside and Pelargonidin 3-O-beta-D-glucoside in the leaves were both very low. Peonidin O-hexoside and Cyanidin 3, 5-O-diglucoside, especially Cyanidin 3, 5-O-diglucoside in the leaves were abundantand, and displayed significant differences at two periods, meaning they may be the key substances for the final color of *A. pseudosieboldianum*.

### Production statistics of sequencing data

In order to understand the molecular mechanism of color change in *A. pseudosieboldianum* leaves in autumn, sequencing was performed using the Illumina Hiseq 2500 (Additional file 1: Table S1). A total of 67.47 Gb of clean data was obtained from these sequencing results, and the percentage of Q30 bases was 93.10 % or more. After assembly, 50,501 unigenes were identified. Among these there were 20,706 unigenes over 1 kb in length, and the error rate of sequencing was less than 0.1 %, which indicates that the quality of sequencing data was good and could be used for subsequent analysis.

### Statistics of sequencing data assembly results

These recombinant sequence dataset yielded 115,413 transcripts and 50,501 unigenes, among which, the N50 (accounting for 50 % of the maximum length nucleotide sequence of all single genes) was 2267 nt and 1979 nt, respectively. There were 17,366 (34.39 %) unigenes between 300 and 500 nt, 23,580 (46.69 %) unigenes between 500 and 2000 nt, and 9,555 (18.92 %) unigenes longer than 2000 nt (Table 1).

**Table 1:** Length distributions of the transcripts and unigenes from *de novo* assembly

Length range	Transcript	Unigene
300–500	24,236(21.00 %)	17,366(34.39 %)
500–1000	26,476(22.94 %)	12,429(24.61 %)
1000–2000	33,114(28.69 %)	11,151(22.08 %)
2000+	31,587(27.37 %)	9555(18.92 %)
Total Number	115,413	50,501
Total Length	179,159,431	62,348,493
N50 Length	2267	1979
Mean Length	1552.33	1234.60

### Functional annotation and classification

Unigene sequence was then compared with gene sequences in the NR, Swiss-Prot GO, COG, KOG, eggNOG 4.5, and KEGG databases using BLAST software (e < 0.00001). 35,316 unigenes were identified, accounting for 70.01 % of the 50,501 unigenes. 12,984 unigenes were annotated in the COG database, 25,375, 12,487 and 19,460 unigenes were annotated in the GO, KEGG, and KOG databases respectively. 25,226 unigenes were annotated in the Pfam database. 19,796 unigenes and 32,498 unigenes were also annotated in the Swanshot and eggNOG databases respectively (Table 2).

**Table 2:** Statistics of comparisons with databases

Anno_ Database	300 < = length < 1000	length > = 1000	Annotated Number
COG_Annotation	4534	8450	12,984
GO_Annotation	11,027	14,348	25,375
KEGG_Annotation	4861	7626	12,487
KOG_Annotation	7536	11,924	19,460
Pfam_Annotation	9281	15,945	25,226
Swissprot_Annotation	6715	13,081	19,796
eggNOG_Annotation	14,214	18,284	32,498
Nr_Annotation	16,192	18,832	35,024
All_Annotated	16,431	18,885	35,316

**Fig. 2:**
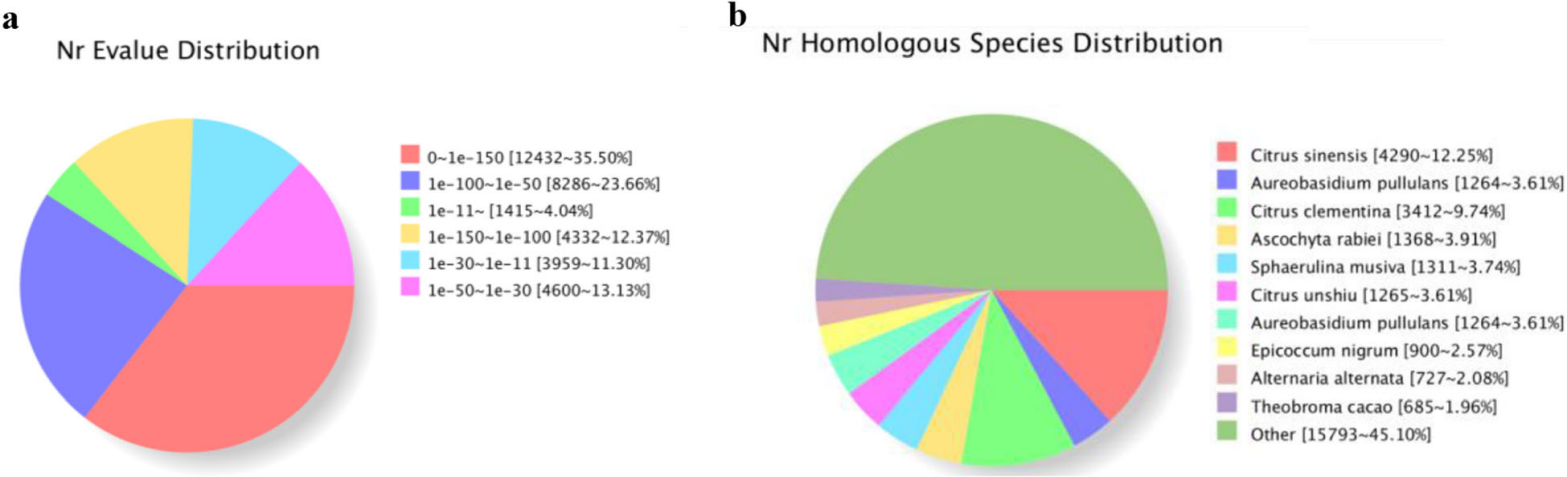
Characteristics of homology search of *A. pseudosieboldianum* unigenes. **a** E-value distribution in the NR database for each unigene. **b** Species taxonomy based on the NR database

According to NCBI NR database and E-value distribution, the number of unigenes annotated in our dataset was 35,024, of which 71.53 % of these unigenes (E < 10 ^− 50^) had strong homology and 47.87 % of these unigenes (E < 10^− 100^) had very strong homology (Fig. 2a).Ten popular-related species were also annotated based on the NCBI NR database (Fig. 2b). The highest homology to *A. pseudosieboldianum* was *Citrus sinensis*, accounting for 12.25 % homology, followed by *Citrus clementina*, which accounted for 9.74 % homology.

**Fig. 3:**
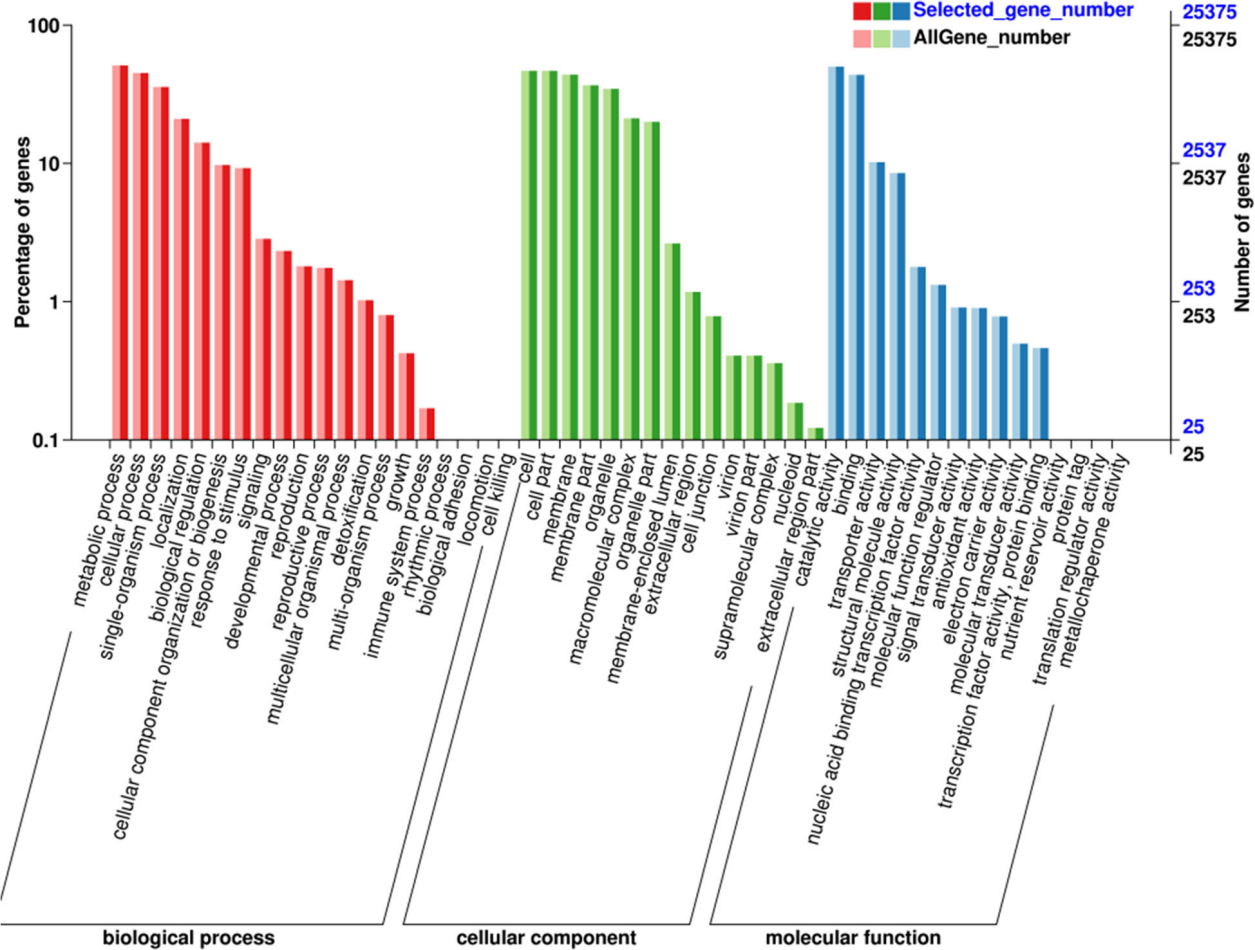
Histogram of GO classification of assembled unigenes

GO databases are divided into three categories: biological process, cellular component and molecular function, which are further divided into 42 functional subgroups. Biological process had the largest number of annotated unigenes, included metabolic process and cellular process with 13,141 (51.78 %) unigenes and 11,546 (45.5 %) unigenes, respectively. The cellular component class mainly included cell and cell part, with 11,886 (46.84 %) unigenes and 11,806 (46.53 %) unigenes, respectively. The molecular function category mainly included catalytic activity and binding, and there were 12,691 (50.01 %) unigenes and 1, 1049 (43.54 %) unigenes (Fig. 3).

In addition, Annotation data about COG and KEGG were found in Additional file 2: Fig. S1 and Additional file 3: Table S2, respecially.

### Differentially Expressed Genes (DEGs)

**Fig. 4:**
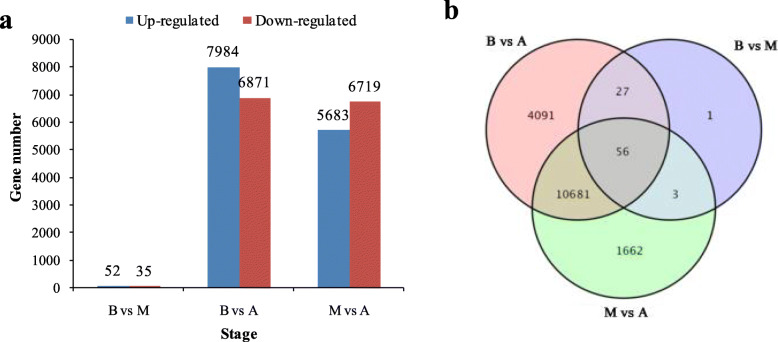
Differentially expressed genes at three stages. **a** The statistics of differentially expressed genes; **b** Venn Diagram result among three stages

In order to explore the genes related to anthocyanin biosynthesis in *A. pseudosieboldianum* at different color-changing stages, the differential expression of *A. pseudosieboldianum* samples at different color-changing stages were then analyzed. The results showed that there were 16,521 DEGs in the three color-changing periods of *A. pseudosieboldianum* (Fig. 4a). Comparing between the early stage (B) and the middle stage (M), there were 87 significant DEGs, with 52 up-regulated and 35 down-regulated. Between with the early stage (B) and the final stage (A), there were 14,855 DEGs, of which 7984 were up-regulated and 6871 were down-regulated. In a comparison of the middle stage (M) and the final stage (A), there were 12,402 DEGs, 5683 up-regulated and 6719 down-regulated, in *A. pseudosieboldianum* (Fig. 4b).

In order to further understand the function of these respective DEGs, we carried out KEGG pathway enrichment analysis in the three stages of *A. pseudosieboldianum*. Our results showed that there were 16,521 differentially expressed genes in the three stages (B, M and A). The anthocyanin biosynthesis pathways related to leaf tone control were significantly enriched in B vs. M and B vs. A up-regulated genes. Phenylalanine metabolic pathways were significantly enriched in B vs. M and B vs. A up-regulated genes (Additional file 4: Table S3; Additional file 5: Table S4).

### Candidate genes involved in the anthocyanin biosynthesis Pathway

**Fig. 5:**
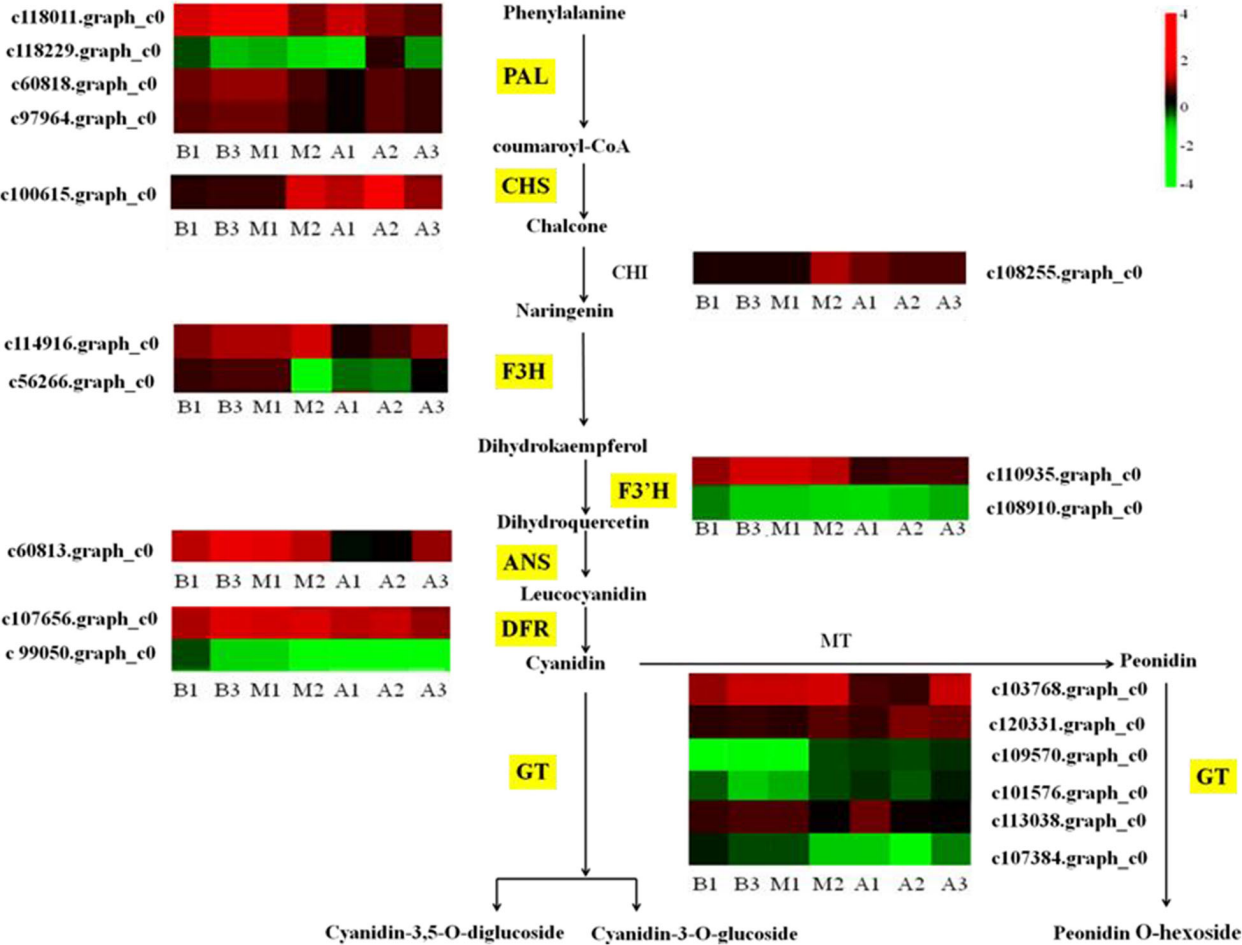
Thermographic analysis of gene pathways related to flavonoid synthesis in *A. pseudosieboldianum* leaves at B, M and A stages. Early stage: B; mid- stage: M; last stage: A. B, M and A are arranged horizontally at all stages and single genes are listed vertically. The annotations are displayed next to the corresponding genes. All FPKM values of single genes are plotted logarithmically

Twenty candidate genes were identified that covered almost all known enzymes involved in anthocyanin biosynthesis. Four PAL genes (c118011.graph_c0, c118229.graph_c0, c60818.graph_c0, c97964.graph_c0), one CHS gene was detected (c100615.graph_c0), one CHI gene (c108255.graph_c0), two F3H genes (c114916.graph_c0, c56266.graph_c0) were detected in the upstream phenylalanine pathway, and two F3’H genes (c110935.graph_c0, c108910.graph_c0), one *ANS* genes, two DFR genes, and six GT genes also detected in the downstream phenylalanine pathway. Combined with contents of metabolites, the gene pathways related to anthocyanin synthesis were analyzed in *A. pseudosieboldianum* (Fig. 5).

### Screening of different transcription factors for anthocyanin biosynthesis

Transcription factors play an important role in plant development and secondary metabolism. In this experiment, we screened out 31 MYB genes, 15 bHLH genes, and 28 WD40 protein genes from the three DEGs of B, M and A stages of *A. pseudosieboldianum*. In the 31 MYB genes, 17 were up-regulated and 14 down regulated (Additional file 6: Table S5). In the 15 bHLH genes, 6 were up-regulated and 9 down regulated. In the 28 WD40 protein genes, 25 were up-regulated and 3 down regulated.

### qRT-PCR confirmation of RNA-seq data

**Fig. 6:**
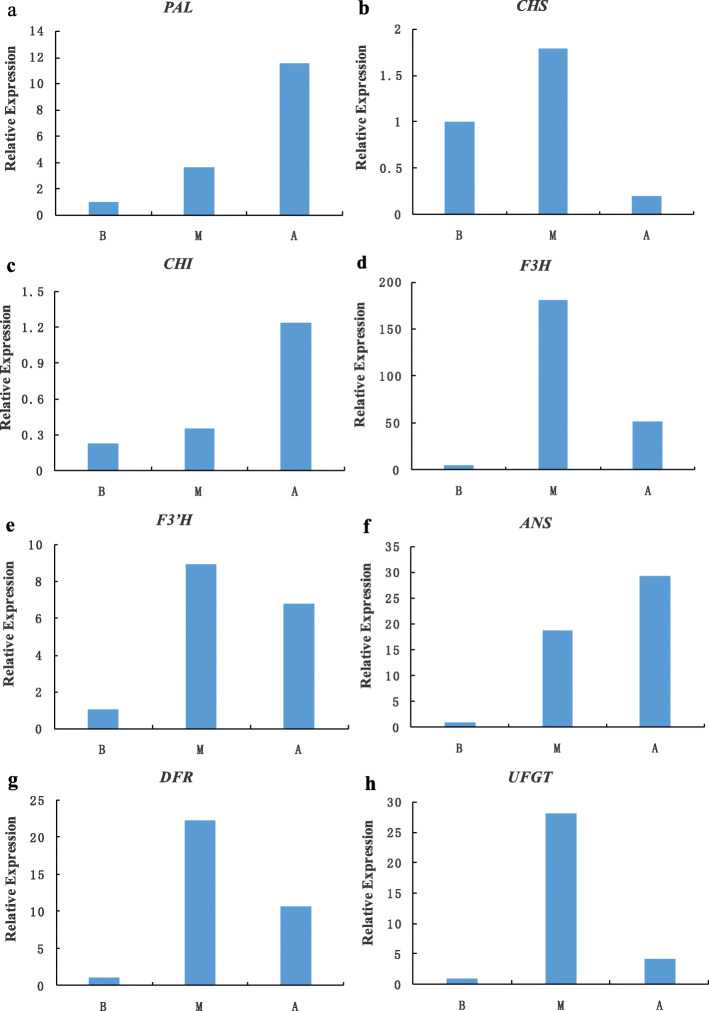
Expression analysis of eight differentially expressed genes related to flavonoid and anthocyanin biosynthesis in *A. pseudosieboldianum*

In order to verify the accuracy of our sequencing data, we selected eight genes involved in anthocyanin biosynthesis, and analyzed the expression level in leaves of different color from these three different stages of *A. pseudosieboldianum* by qRT- PCR. The results showed that all of these selected genes had similar expression patterns than identified in the RNA sequencing data (Fig. 6). Therefore, the data obtained in our study can be used to analyze the anthocyanin biosynthesis and metabolism gene in *A. pseudosieboldianum*.

## Discussion

*A. pseudosieboldianum* is a wild ornamental maple native to Northeast China. Like *A. palmatum* Thunb., *A. pseudosieboldianum* belongs to Sect. Palmata Paxand Ser. Palmata (Pax) Pojark. There were many cultivars of *A. palmatum* and they had strong ecological adaptability [27]. However, there are few varieties of *A. pseudosieboldianum*, which was still in the wild state or in scenic forests, and are rarely used in urban greening even if the maple leaves are red and beautiful in autumn and have high ornamental value.

At present, transcriptome sequencing technology has been used to study vegetables color formation [28], flower color mechanisms [10, 29], fruit development [30, 31]. Some scholars have analyzed the color mechanism of the related species in *Acer* [32]. However, due to the lack of genomic reference sequences, the molecular mechanism of leaf color is difficult to decipher in *A. pseudosieboldianum*. The change of anthocyanin content in plants was shown to be related to the differential expression of key genes encoding structural enzymes in the anthocyanin biosynthesis pathway [10]. The different genes including *PAL*, *CHS*, *ANS*, *UFGT*, *FLS*, *C4H*, *4CL*, *DFR* and *ANR* were identified in the flavonoid biosynthesis pathway from the purple bud tea plant by transcriptome sequencing [33]. In this study, we used transcriptome sequencing technology to sequence and compare three different coloring stages of *A. pseudosieboldianum* leaves in autumn. We detected four *PAL*, one *CHS*, one *CHI*, two *F3H*, two *F3’H*, one *F3’5’H*, two *DFR*, one *ANS*, and six *UFGT* genes in the flavonoid anthocyanin complex related to leaf color in *A. pseudosieboldianum*. Three *GT* genes were down regulated in the M vs. A stage, which indicated that the change of leaves from green to red was controlled by multiple single genes.

Both *F3’H* and *F3’5’H* belong to the cytochrome P450 superfamily [34]. *F3’H* is an important intermediate in the synthesis of cyaniding, and *F3’5’H* is a key enzyme in the synthesis of blue flower anthocyanin. Masukawa T [35] reported that *F3’H* could make red cyanidin accumulate in purple and red root radishes. *F3’5’H* mainly accumulated in the blue waterlily [11]. Many important flower crops can’t produce turquoise, meaning they cannot appear blue. In this study, two *F3’H* and one F3’5’H were detected, but the expression of *F3’5’H* was very small, which may be caused leaf colour did not appear blue.

*DFR* is the key enzyme that catalyzes the conversion of dihydroflavonol to corresponding colorless geranium delphinium and cyanidin [36]. The main function of *ANS* is to oxidize colorless proanthocyanidins to produce colored anthocyanidins, which are the first colored compound in the anthocyanin synthesis pathway [37]. *ANS* was originally identified in a maize A2 mutant and cloned by the transposon tagging technique [38]. *GT* is mainly responsible for transforming unstable anthocyanins into stable anthocyanins. Studies have shown that the expression of *UFGT* is different in different varieties [39]. For example, anthocyanin accumulation in apple was positively correlated with *UFGT* activity. The change of *UFGT* activity in grape leads to the change of their phenotype from white to red [40]. In this study, *UFGT* (c103768.graph_c0) expression in *A. pseudosieboldianum* leaves first accumulated and then was consumed in the process of leaf color formation, which was consistent with the conclusion that *UFGT* consumption was needed for paeoniae anthocyanin synthesis.

At present, the research about anthocyanin biosynthesis structural genes has been gradually improved, and the research on transcription factors has become the focus. The transcription factors may also be one of the important indicators of causing *A. pseudosieboldianum* to turn green and red. Now MYB transcription factors for anthocyanin biosynthesis have been identified and isolated in many plants. Some studies have shown that MYB transcription factor can enhance or inhibit some aspects of regulation [10]. It was found that *PqMYB113* was a transcription factor promoting anthocyanin synthesis in the leaves of peony, while *PqMYB4* was a transcription factor inhibiting anthocyanin synthesis in the leaves of peony. In this study, we found that *ApMYB4* gene was down-regulated in the stage of green to red transformation, which is consistent with the previous research results, indicating that *ApMYB4* gene may be a transcription factor promoting anthocyanin synthesis in *A. pseudosieboldianum.*

It is worth mentioning that Rosinidin O-hexoside was found in *A. pseudosieboldianum* leaves, although the content was very small. There was no Rosinidin O-hexoside found in *Acer* in previous studies. The distribution of Rosinidin O-hexoside in plants is very limited and has only been reported in *Catharanthus roseus* [41] and *Primula* [12]. In addition, the cyanidin 3-glucoside contents could be used as a quantitative index to determine the color of *Acer palmatum ‘atropurpureum’* [42]. It was also found that the leaf color changing from green to red in *A. palmatum* was the result of the increase of the mass fraction of cyanidin galactoside and the decrease of the mass fraction of chlorophyll [43]. In this study, the contents of differential metabolites were very high about Cyanidin 3, 5-O-diglucoside. The above research results revealed that Cyanidin was important anthocyanin in *Acer*, and played a key role of leaf color change in autumn. This study provided the basis for molecular breeding theory for ornamental plant leaf color improvement.

## Conclusions

In this study, five anthocyanins were detected in the leaves of *A. pseudosieboldianum*, especially, Cyanidin 3, 5-O-diglucoside played an important role for the final leaf color. A total of 50,501 unigenes were produced at three stages of leaf color changing among which 16,521 DEGs and 64 unigenes were identified as color-related homologous genes. Four *PAL*, one *CHS*, one *CHI*, two *F3H*, two *F3’H*, one *F3’5’H*, two *DFR*, one *ANS* and six *GT* about anthocyanin synthesis pathway were detected. Combined with the detection of metabolites, the gene pathways related to anthocyanin synthesis were conducted. Also, related regulatory genes include MYB, bHLH, and WD40 were found. This study provides a theoretical basis for the formation of leaf color in *A. pseudosieboldianum*.

## Methods

### Plant materials and treatments

**Fig. 7:**
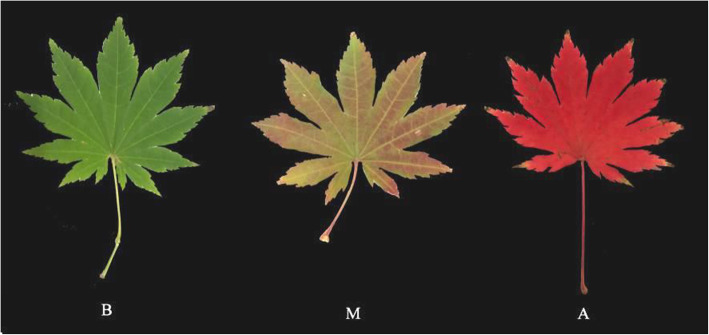
The three stages of color change in *A. pseudosieboldianum* leaf. **b**: Initial stage, the leaves were all green. **m**: Mid stage, the leaves were both red and green. **a**: Last stage, the leaves were all red

The materials tested were *A. pseudosieboldianum* plants that were five years old from Tianchi Square, Yanji City, Jilin Province (The plant materials were identified by Professor Liu Ji-Sheng from Agriculture college, Yanbian University, engaged in dendrological research for many years). Three leaf samples were collected separately at three different stages (early stage: B; mid- stage: M; last stage: A) with three replicate libraries per stage (Fig. 7). Three stages were September 21, 2018 (B) September 30, 2018 (M) and October 11, 2018 (A). All flesh samples were frozen immediately in liquid nitrogen, and then stored in a refrigerator at -80 ℃ for transcriptome sequencing and anthocyanin metabolite analysis.

### Extraction identification and data analysis of anthocyanin metabolites

Leaf tissue samples of *A. pseudosieboldianum* were ground to a powder, and 100 mg of powder was dissolved in 1.0 ml extract solution (70 % methanol aqueous solution). The dissolved sample was placed in a refrigerator overnight at 4 ℃, and then vortexed three times during the period to improve the extraction rate. After centrifugation, the supernatant was reserved and the sample was filtered with a microporous filter membrane, and then stored in a sample bottle for LC-MS / MS analysis. We used multiple reaction monitoring (MRM) for qualitative and quantitative analysis of metabolites by mass spectrometry. Combining single variable statistical analysis and multivariate statistical analysis, we calculated the fold-change, and called a metabolite as a differential metabolite when its value was between a fold change ≥ 2 and a fold change ≤ 0.5. The differential metabolites were then annotated using the KEGG database [44].

### RNA isolation library construction and RNA-Seq

Total RNA was extracted from leaf samples using an RNA extraction kit (Beijing Tiangen in China). Agarose electrophoresis and the Agilent 2100 Bioanalyzer were used to determine the concentration, purity and integrity of RNA samples. Then, PolyA mRNA was reverse transcribed into cDNA, and the construction and sequencing of the cDNA library was completed by the BMK Technology company in Beijing. Raw reads were obtained using an Illumina Hiseq 2500 sequencing platform, and after filtering, clean reads were obtained. Contigs were assembled by overlapping information between sequences, transcripts were locally assembled, and unigenes were obtained by homologous clustering and splicing of transcripts with Tgicl and Phrap software, respectively [45].

### De novo assembly and functional annotation

After obtaining high quality sequencing data, it was necessary to assemble the genomic sequence of *A. pseudosieboldianum*. First, Trinity software parsed the sequencing reads into shorter fragments (K-mers), extends these fragments into longer fragments (Contig), and uses the overlap between these fragments to determine the fragment set (Component). Finally, using the dual methods of De Bruijn mapping and sequencing read information analysis, each transcript sequence was identified in each fragment set. The Unigene sequence was compared with the gene sequence in NR [46], Swiss-Prot [47], GO [48], COG [49], KOG [50], eggNOG4.5 [51], KEGG database by Blast software [52] (e < 0.00001). Using KOBAS 2.0 [53], the KEGG orthology result of unigenes from KEGG was obtained, and after predicting the amino acid sequence of each unigene, we used HMMER [54] software to compare with the Pfam [55] database, select unigenes whose BLAST parameter E-values were not greater than 1e^− 5^ and whose HMMER parameter E-values were not more than 1e^− 10^, and thus, finally obtained a unigene with annotation information.

### Expression and differentially expressed unigene annotation

Bowtie [56] was used to compare the sequenced reads with a unigene library, and RSEM [57] was used to estimate the expression level. The expression abundance of each corresponding unigene was expressed by its FPKM [58] value. It is a common method for estimating gene expression level in transcriptome sequencing data analysis. The use of FPKM values can eliminate the influence of gene length and sequencing on calculations of gene expression. When detecting differentially expressed genes, DESeq2 was used to analyze the differentially expressed genes between the sample groups and the differentially expressed gene sets between two different conditions were identified. In the process of differential expression analysis, the Benjamini−Hochberg method was used to correct the significance p-value of the original hypothesis test, so as to reduce the false positives in independent statistical hypothesis testing for a large number of gene expression values. In the screening process, the criterion was that the FDR (False Discovery Rate) was less than 0.01 and the difference factor FC (Fold Change) was greater than or equal to 2. Between these two factors, the FC represented the ratio of expression between two samples (groups).

### Gene validation and expression analysis

In order to validate our differential gene expression analysis, RT-qPCR was used to validate the differentially expressed genes related to anthocyanin biosynthesis [59]. We used a fluorescence quantitative Kit (2×SYBR ® green premix) and an analytikjena-qTOWER 2.2 fluorescence quantitative PCR instrument for quantitative analysis. The primer sequences can be found in Additional file 7: Table S6. The reaction procedure was as follows: 95 ℃ for 3 min, 95 ℃ for 10 s, 58 ℃ for 30 s, for a total of 39 cycles. Melt curve analysis (60 ℃ ~ 95 ℃ +1 ℃ / cycle, holding time 4 s), and carried out centrifugation on PCR plate centrifuge at 4 ℃ 6000 rpm for 30 s. Finally, we put it in quantitative PCR for amplification, using c110191.graph_c0 as an internal reference gene.

### Statistical analysis

The procedure was repeated three times for each sample and the relative expressions were calculated using the 2^−∆∆Ct^ method. Excel and GraphPad Prism 5 were used for chart preparation. The R-3.4.2 was used to conduct the heatmap.

## Supplementary Information

**Additional file 1: Table S1.** Statistics of sequencing data across the nine libraries in *A. pseudosieboldianum*.

**Additional file 2: Figure S1. **COG classifications of annotated unigenes.

**Additional file 3: Table S2. **KEGG pathway annotation in *A. pseudosieboldianum* unigenes.

**Additional file 4: Table S3. **KEGG pathway enrichment analysis of DEGs between B and M.

**Additional file 5: Table S4.** KEGG pathway enrichment analysis of DEGs between B and A.

**Additional file 6: Table S5. **MYBs identified in differentially expressed genes.

**Additional file 7: Table S6.** Designed primers for RT-qPCR.

## Data Availability

Raw-reads data were deposited in the NCBI Sequence Read Archive (SRA) with accession number of PRJNA596335. The Transcriptome Shotgun Assembly project has been deposited at DDBJ/EMBL/GenBank under the accession GJBB00000000.

## Abbreviations

HPLC: High-performance liquid chromatography
ESI-MS/MS: Electrospray ionization tandem mass spectrometry
COG: Clusters of orthologous groups
Go: Gene Ontology
NCBI: The US National center for biotechnology information
NR: Non-redundant protein sequence database
KOG: Clusters of orthologous groups for eukaryotic complete genomes
PAL: Phenylalanine ammonia-lyase
CHS: Chalcone synthase
CHI: Chalcone isomerase
F3H: Flavone 3-hydroxylase
F3’H: Flavonoid 3’-hydroxylase
F3’5’H: Flavonoid 3’5’-hydroxylase
DFR: Dihydroflavonol reductase
ANS: Anthocyanidin synthase
GT: Glucosyltransferase
eggnog: Evolutionary genealogy of genes:Non-supervised Orthologous Groups
KEGG: Kyoto Encyclopedia of Genes and Genomes
DEG: Differentially expressed genes
MT: Methyltransferase
qRT-PCR: Quantitative real-time reverse transcription PCR

## References

[1] Dai SL, Huang H, Fu JX, Hong Y (2013). Advances in molecular breeding of ornamental plants. Chinese Bull Bot.

[2] Sjöman H, Hirons AD, Bassuk NL (2015). Urban forest resilience through tree selection-variation in drought tolerance in *Acer*. Urban For Urban Gree.

[3] Li XM. Physiological characters of *Pyracantha fortuneana* ‘Harlequin’ leaves during color-changing period in autumn and winter. J. Shanghai Jiaotong U. (Agr Sci). 2013; 31: 82–7.

[4] Ren J, Chen Z, Tang F, Xuan Y, Yang F, Lu XY, Fu SL (2019). Study on leaf color related chemicals components based on comparing *Acer rubum* L. cv.‘Yanhong’ and ‘Jinseqiutian’. J Anhui Agr U.

[5] Li YK, Fang JB, Qi XJ, Lin MM, Zhong YP, Sun LM, Cui W (2018). Combined analysis of the fruit metabolome and transcriptome reveals candidate genes involved in flavonoid biosynthesis in *Actinidia arguta*. Int J Mol Sci.

[6] Song XW, Wei XB, Di SK, Pang YZ (2019). Recent advances in the regulation mechanism of transcription factors and metabolic engineering of anthocyanins. Chinese Bull Bot.

[7] Hu JT, Chen GP, Zhang YJ, Cui BL, Yin WC, Yu XH, Zhu ZG (2015). Anthocyanin composition and expression analysis of anthocyanin biosynthetic genes in kidney bean pod. Plant Physio Bioch.

[8] Li BB, Hou ZX, Yang JF, Chen L, Wan RM (2018). Variations of flavonoids and soluble sugars in ‘Northland’ blueberry leaf during the color changing process. J Agr Sci Technol.

[9] Feng FJ, Li MJ, Ma FW, Cheng LL (2013). Phenylpropanoid metabolites and expression of key genes involved in anthocyanin biosynthesis in the shaded peel of apple fruit in response to sun exposure. Plant Physiol Bioch.

[10] Zhang HS, Tian H, Chen MX, Xiong JB, Cai H, Liu Y (2018). Transcriptome analysis reveals potential genes involved in flower pigmentation in a red-flowered mutant of white clover (*Trifolium repens* L.). Genomics.

[11] Wu Q, Wu J, Li SS, Zhang HJ, Feng CY, Yin DD, Wu RY (2016). Transcriptome sequencing and metabolite analysis for revealing the blue flower formation in waterlily. BMC Genom.

[12] Valentina S, Maja MP, Franci S, Vlasta C (2017). Phenolic accumulation in hybrid primrose and pigment distribution in different flower segments. J Am Soc Hortic sci.

[13] Jia ZD, Ma PY, Bian XF, Yang Q, Guo XD, Xie YZ (2014). Biosynthesis metabolic pathway and molecular regulation of plants anthocyanin. Acta Bot Boreal-Occident Sin.

[14] Wang W, Zheng W, Xu XD, Chen J, Wang TX (2017). Coloring mechanism analysis of mosaic leaves in *Camellia reticulata* Lindl. based on sequencing of transcriptome. Acta Bot Boreal Occident Sin.

[15] Duan YJ, Zhang LG, He Q, Zhang MK, Shi JC (2012). Expression of transcriptional factors and structural genes of anthocyanin biosynthesis in purple-heading Chinese Cabbage. Acta Hortic Sin.

[16] Liu XF, Li F, Yin XR, Xu CJ, Chen KS (2013). Recent advances in the transcriptional regulation of anthocyanin biosynthesis. Acta Hortic Sin.

[17] Shi Q, Li X, Du J, Li X (2019). Anthocyanin synthesis and the expression patterns of bHLH transcription factor family during development of the Chinese jujube fruit (*Ziziphus jujuba* Mill.). Forests.

[18] Grace SC, Logan BA, Adams WW (1998). Seasonal differences in foliar content of chlorogenic acid a phenylpropanoid antioxidant in Mahonia repens. Plant Cell Environ.

[19] Zhang X, Xiao TT, Li J, Wang YT, Liu GL (2016). Effects of water stress on the growth and leaf color in *Acer rubrum*. Jiangsu Agricultural Sciences.

[20] Oberbauer SF, Starr G (2002). The role of anthocyanins for photosynthesis of alaskan arctic evergreens during snowmelt. Adv Bot Res.

[21] Sun B, Zheng DC, Cui HM (2009). Leaf color change in *Acer pseudo-sieboldianum* in autumn. J Northeast For U.

[22] Pang QY, Zhuo LH (2007). Spatial difference in physiological indexes of autumn colored maple leaves. J Northeast For U.

[23] Qu X, Sun B, Yang YH (2012). Leaf color performance and its application of introduced *A. pseudosieboldianum*. Terrttory Nat Resour Study.

[24] Xian Y, Dong X, Xie XM, Wu D, Han B, Wang Y (2019). Effect of conservation conditions on restricting conservation of *Acer rubrum* cv. ‘Somerset’. Chinese Bull Bot.

[25] Wang YS, Wang H, Fan ZY, Chen Y, Jin YF, Gao ML (2020). Identifying genes associated with leaf color in kale (Brassica oleracea L. var. acephala DC.) based on transcriptome analysis. Genom Appl Biol.

[26] Jiang Y, Wang Q, Shen QQ, Zhuo BP, He JR (2020). Transcriptome analysis reveals genes associated with leaf color mutants in *Cymbidium longibracteatum*. Tree Genetics Genomes.

[27] Rong LP, Li QZ, Li SS, Tang L, Wen J (2016). De novo transcriptome sequencing of *Acer palmatum* and comprehensive analysis of differentially expressed genes under salt stress in two contrasting genotypes. Mol Genet Genom.

[28] Kodama M, Brinch-Pedersen H, Sharma S, Holme IB, Joernsgaard B, Dzhanfezova T, Amby DB (2018). Identification of transcription factor genes involved in anthocyanin biosynthesis in carrot (*Daucus carota* L.) using RNA-SEq. BMC Genomics.

[29] Qu Y, Ou Z, Yang FS, Wang S, Peng JS (2018). The study of transcriptome sequencing for flower coloration in different anthesis stages of alpine ornamental herb (*Meconopsis* ‘Lingholm’). Gene.

[30] Fang Z, Zhou DR, Ye XF, Jiang CC, Pan SL (2016). Identification of candidate anthocyanin-related genes by transcriptomic analysis of ‘Furongli’ *Plum* (*Prunus salicina* Lindl.) during fruit ripening using RNA-SEq. Front. Plant Sci.

[31] Shi LY, Chen X, Chen W, Zheng YH, Yang ZF (2018). Comparative transcriptomic analysis of white and red Chinese bayberry (*Myrica rubra*) fruits reveals flavonoid biosynthesis regulation. Sci Hortic.

[32] Chen Z, Lu XY, Xuan Y, Tang F, Wang JJ, Shi D, Fu SL (2019). Transcriptome analysis based on a combination of sequencing platforms provides insights into leaf pigmentation in *Acer rubrum*. BMC Plant Biol.

[33] Jiang HB, Xia LF, Tian YP, Dai WD, Sun YN, Chen LB (2018). Transcriptome analysis of anthocyanin synthesis related genes in purple bud tea plant. J Plant Genet Resour.

[34] Rosati C, Cadic A, Duron M, Ingouff M, Simoneau P (1999). Molecular characterization of the anthocyanidin synthase gene in Forsythis×intermedia reveals organ-specific expression during flower development. Plant Sci.

[35] Masukawa T, Cheon KS, Mizuta D, Kadowaki M, Nakatsuka A, Kobayashi N (2019). Development of mutant RsF3′H allele-based marker for selection of purple and red root in radish (*Raphanus sativus* L.var. *longipinnatus* L.H.Bailey). Euphytica.

[36] Holton TA, Cornish EC (1995). Genetics and biochemistry of anthocyanin biosynthesis. Plant Cell.

[37] Springob K, Nakajirna J, Yamazaki M, Saito K (2003). Recent advances in the biosynthesis and accumulation of anthocyanins. Nat Prod Rep.

[38] Menssen A, Hohmann S, Martin W, Schnable PS, Peterson PA, Saedler H, Gierl A (1990). The En/Spm transposable element of Zea mays contains splice sites at the temini generating a novel intron from a dSpam element in the A2 gene. Embo J.

[39] Li JC, Li TH, Wang ZG, Li BJ (2010). Cloning and expression of UFGT gene in skin of max red Bartlett. Acta Botanica Boreali-Occidentalia Sinica.

[40] Kobayashi S, Ishimaru M, Ding CK, Yakushiji H, Goto N (2001). Comparison of UDP-glucose: flavonoid 3-O-glucosyltransferase(UFGT) gene sequences between white grapes (*Vitis vinifera*) and their sports with red skin. Plant Sci.

[41] Toki K, Saito N, Irie Y, Tatsuzawa F, Shigihara A, Honda T (2008). 7-O-Methylated anthocyanidin glycosides from *Catharanthus roseus*. Phytochemistry.

[42] Schmitzer V, Stampar F, Veberic R, Osterc G (2009). Phase change modifies anthocyanin synthesis in *Acer palmatum* Thunb. (Japanese maple) cultivars. Acta Physiol Plant.

[43] Cai XY, Li HH, Li L, Yu H, Chen G, Bao NNDT (2015). Pigment composition and leaf color change in *Acer palmatum*. J Northeast For U.

[44] Kanehisa M, Goto S, Kawashima S, Okuno Y, Hattori M (2004). The KEGG resource for deciphering the genome. Nucleic Acids Res.

[45] Grabherr MG, Haas BJ, Yassour M, Levin JZ, Thompson DA, Amit I, Adiconis X (2011). Full-length transcriptome assembly from RNA-Seq data without a reference genome. Nat Biotechnol.

[46] Deng YY, Li JQ, Wu SF, Zhu YP, Chen YW, He FC (2006). Integrated nr database in protein annotation system and its localization. Comput Eng.

[47] Apweiler R, Bairoch A, Wu CH, Barker WC, Boeckmann B, Ferro S, Gasteiger E (2004). UniProt: the Universal Protein knowledgebase. Nucleic Acids Res.

[48] Ashburner M, Ball CA, Blake JA, Botstein D, Butler H, Cherry JM, Davis AP (2000). Gene ontology: tool for the unification of biology. Nat Genet.

[49] Tatusov RL, Galperin MY, Natale DA, Koonin EV (2000). The COG database: a tool for genome scale analysis of protein functions and evolution. Nucleic Acids Res.

[50] Koonin EV, Fedorova ND, Jackson JD, Jacobs AR, Krylov DM, Makarova KS, Mazumber R (2004). A comprehensive evolutionary classification of proteins encoded in complete eukaryotic genomes. Genome Biol.

[51] Huerta-Cepas J, Szklarczyk D, Forslund K, Cook H, Heller D, Walter MC, Ratteri T (2016). eggNOG 4.5: a hierarchical orthology framework with improved functional annotations for eukaryotic prokaryoticand viral sequences. Nucleic Acids Res.

[52] Altschul SF, Madden TL, Schaffer AA, Zhang J, Zhang Z, Miller W, Lipman DJ (1997). Gapped BLAST and PSI BLAST: A newgeneration of protein database search programs. Nucleic Acids Res.

[53] Xie C, Mao XZ, Huang JJ, Ding Y, Wu JM, Dong S, Kong L (2011). KOBAS 2.0: a web server for annotation and identification of enriched pathways and diseases. Nucleic Acids Res.

[54] Eddy SR (1998). Profile hidden Markov models. Bioinformatics.

[55] Finn RD, Bateman A, Clements J, Coggill P, Eberhardt RY, Eddy SR, Heger A (2014). Pfam: the protein families database. Nucleic Acids Res.

[56] Langmead B, Pop M (2009). Ultrafast and memory-efficient alignment of short DNA sequences to the human genome. Genome Biol.

[57] Li B, Dewey CN (2011). RSEM: accurate transcript quantification from RNA-Seq data with or without a reference genome. BMC Bioinformatics.

[58] Trapnell C, Williams BA, Pertea G, Mortazavi A, Kwan G, Baren MJ, Salzberg SL (2010). Transcript assembly and quantification by RNA seq reveals unannotated transcripts and isoform switching during cell differentiation. Nat Biotechnol.

[59] Pfaffl MW (2001). A new mathematical model for relative quantification in real-time RT-PCR. Nucleic Acids Res.

