# Supplementary material for: De novo transcriptome sequencing and anthocyanin metabolite analysis reveals leaf color of Acer pseudosieboldianum in autumn
Source: BMC Genomics. 2021 May 25;22:383. doi: 10.1186/s12864-021-07715-x (PMC8145822; doi:10.1186/s12864-021-07715-x)
Supplement: Supplementary file 1 — Additional file 1: Table S1. Statistics of sequencing data across the nine libraries in A. pseudosieboldianum. [file 12864_2021_7715_MOESM1_ESM.docx]

**Table S1.** Statistics of sequencing data across the nine libraries in *A. pseudosieboldianum*

| Sample ID | Clean  number | Base number | GC contment | %≥Q30 | Mapped reads | Mapped ratio |
| --- | --- | --- | --- | --- | --- | --- |
| B1 | 24,891,319 | 7,411,181,104 | 45.12% | 94.12% | 20,710,424 | 83.20% |
| B2 | 24,754,322 | 7,373,450,408 | 45.72% | 94.25% | 20,509,397 | 82.85% |
| B3 | 26,344,753 | 7,839,350,686 | 44.02% | 93.10% | 21,123,549 | 80.18% |
| M1 | 20,586,684 | 6,140,849,238 | 44.61% | 94.04% | 17,004,609 | 82.60% |
| M2 | 24,264,520 | 7,220,440,952 | 45.08% | 94.33% | 20,030,805 | 82.55% |
| M3 | 20,951,279 | 6,256,334,358 | 44.34% | 93.99% | 17,134,598 | 81.78% |
| A1 | 26,611,490 | 7,923,701,850 | 44.84% | 94.03% | 21,802,156 | 81.93% |
| A2 | 28,672,411 | 8,529,990,156 | 44.95% | 93.73% | 23,549,563 | 82.13% |
| A3 | 29,433,309 | 8,770,181,170 | 45.13% | 93.44% | 24,196,882 | 82.21% |
