# Supplementary figures and images for: De novo transcriptome sequencing and anthocyanin metabolite analysis reveals leaf color of Acer pseudosieboldianum in autumn
Source: BMC Genomics. 2021 May 25;22:383. doi: 10.1186/s12864-021-07715-x (PMC8145822; doi:10.1186/s12864-021-07715-x)

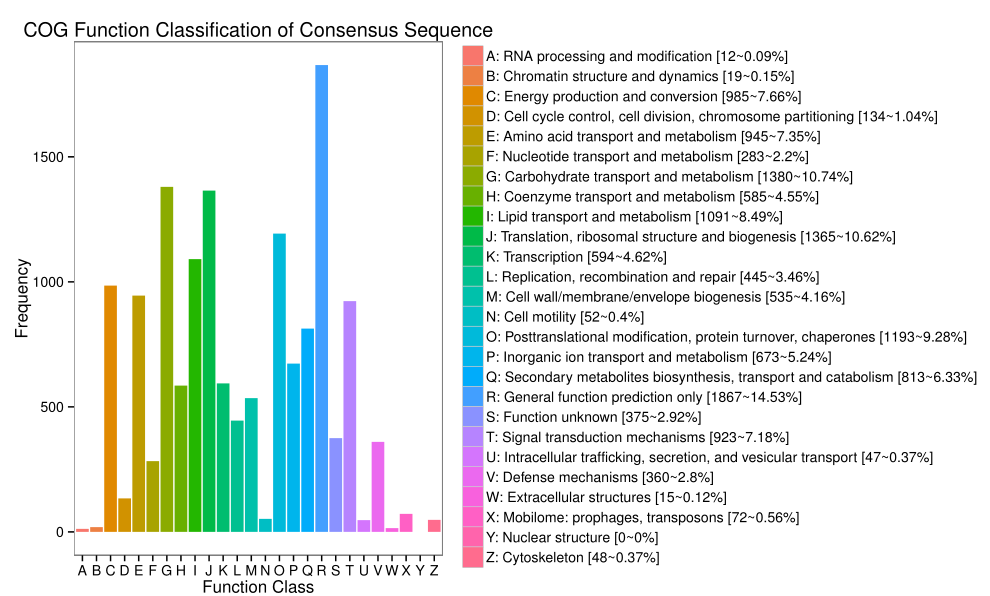


**Fig. S1** COG classiﬁcations of annotated unigenes

Supplement: Supplementary file 2 — Additional file 2: Figure S1. COG classifications of annotated unigenes. [file 12864_2021_7715_MOESM2_ESM.docx]
