# Supplementary material for: De novo transcriptome sequencing and anthocyanin metabolite analysis reveals leaf color of Acer pseudosieboldianum in autumn
Source: BMC Genomics. 2021 May 25;22:383. doi: 10.1186/s12864-021-07715-x (PMC8145822; doi:10.1186/s12864-021-07715-x)
Supplement: Supplementary file 6 — Additional file 6: Table S5. MYBs identified in differentially expressed genes. [file 12864_2021_7715_MOESM6_ESM.docx]

**Table S5. MYBs identified in differentially expressed genes**

| Gene ID | Fold=log2Ratio | Regulated | NR annotation |
| --- | --- | --- | --- |
| c109244.graph_c0 | -1.521742433 | down | L10-interacting MYB domain-containing protein isoform X2 [*Citrus clementina*] |
| c107186.graph_c0 | -2.351636714 | down | Predicted: myb-related protein A isoform X4 [*Citrus sinensis*] |
| c102076.graph_c0 | -5.671477731 | down | Predicted: myb family transcription factor APL isoform X1 [*Citrus sinensis*] |
| c61129.graph_c1 | 4.052683637 | up | Myb domain protein 84 [*Theobroma cacao*] |
| c105871.graph_c0 | -4.122754543 | down | Predicted: transcription factor MYB12 [*Theobroma cacao*] |
| c56626.graph_c0 | 2.661997301 | up | Transcription factor MYB52-like [*Durio zibethinus*] |
| c110899.graph_c1 | 2.148119645 | up | Transcription factor MYB3R-1 isoform X3 [*Citrus clementina*] |
| c109863.graph_c0 | 1.286655164 | up | Putative R2R3-Myb transcription factor [*Citrus sinensis*] |
| c109516.graph_c0 | -1.623630774 | down | Myb/SANT-like domain containing protein [*Trema orientalis*] |
| c103921.graph_c1 | 1.526153568 | up | Lowqualityprotein: myb-related protein 2-like [*Carica papaya*] |
| c116342.graph_c1 | 2.616840482 | up | Myb-like HTH transcriptional regulator family protein, putative [*Theobroma cacao*] |
| c102313.graph_c0 | 2.690702981 | up | Transcription factor MYB73 [*Citrus clementina*] |
| c89887.graph_c0 | 4.237033545 | up | Predicted: myb-related protein Myb4-like [*Nicotiana tomentosiformis*] |
| c91905.graph_c0 | 3.945447764 | up | transcription factor MYB108-like [*Durio zibethinus*] |
| c117758.graph_c0 | -1.723574086 | down | Predicted: myb-binding protein 1A [*Citrus sinensis*] |
| c100815.graph_c0 | -3.268684768 | down | Predicted: myb-like protein Q isoform X1 [*Fragaria vesca subsp. vesca*] |
| c106582.graph_c1 | -4.495528431 | down | L10-interacting MYB domain-containing protein [*Citrus clementina*] |
| c84535.graph_c0 | -2.679472132 | down | Predicted: myb-like protein B [*Vitis vinifera*] |
| c113871.graph_c3 | -3.787570239 | down | Myb-related protein 308-like [*Quercus suber*] |
| c117528.graph_c2 | 3.798032382 | up | Myb_DNA-binding domain-containing protein, partial [*Cephalotus follicularis*] |
| c80395.graph_c0 | 1.127510554 | up | Predicted: myb-related protein 308-like [*Citrus sinensis*] |
| c93803.graph_c0 | 5.450081637 | up | Putative Myb family transcription factor At1g14600 isoform X1 [*Herrania umbratica*] |
| c92727.graph_c0 | 3.009004104 | up | Transcription factor MYB52-like [*Durio zibethinus*] |
| c112345.graph_c0 | -2.634681155 | down | L10-interacting MYB domain-containing protein-like [*Prunus avium*] |
| c111178.graph_c0 | 3.582408767 | up | Transcription factor MYB14-like [*Quercus suber*] |
| c99512.graph_c0 | -3.873217098 | down | MYB-like transcription factor ETC3 [*Durio zibethinus*] |
| c104940.graph_c1 | 1.890670935 | up | Transcription factor MYBS3-like isoform X2 [*Quercus suber*] |
| c117403.graph_c1 | 3.769030868 | up | MYB1 [*Litchi chinensis*] |
| c80367.graph_c1 | -3.674644068 | down | Predicted: myb-related protein 306-like [*Citrus sinensis*] |
| c101462.graph_c0 | -2.308258801 | down | Myb family transcription factor PHL5-like isoform X2 [*Quercus suber*] |
| c119165.graph_c0 | 1.507977474 | up | Predicted: transcription factor MYB1R1 [*Citrus sinensis*] |
